# The London Hospital "Gazette."

**Published:** 1919-08-02

**Authors:** 


### The London Hospital "Gazette."

### The "Gazette" Itself Again.

The once familiar cover of the London Hospital *Gazette*, designed by Mr. Tonks, has again been taken into use, and the readers renew their acquaintance with the jester with the skull, who assumes an "Alas! poor Yorick" attitude and expression. The July number is an excellent one, and includes as insets a caricature of Mr. Openshaw in military garb, and a portrait of the late Mr. Frederick J. Smith and his favourite dog. We learn that pre-war life at the London is renewing fast, khaki is becoming relatively rare, and the clubs are blossoming forth anew. Amongst the many good things of the current number are articles on "Surgery at the London in 1852," "Bygone members of the Hospital Staff," and "Medical Work among the Arabs of Mesopotamia," the last two articles being illustrated. One of the pleasantest phases of peace to an institution is the gathering together of fellow-workers. At the London nurses are returning, preferring palatial bedrooms within brick walls to tiny cubicles in wooden huts; clerks and dressers are seen again in the wards, and the College is a busy hive of industry. As the *Gazette* Editorial says, there is room for all, room for every one of the best brains and the best physiques of the land.

### Early Memories of Miss Luckes.

The current number of the London Hospital *Gazette* publishes a letter from Sir Frederick Treves to Viscount Knutsford in which are described nursing conditions in hospitals in 1880, when nursing everywhere was unorganised, untaught, squalid and heartless, and the career of a nurse was hardly one for a respectable woman. The majority of the nurses were middle-aged or old; a young nurse was almost unknown, for nursing was a calling for the derelict. The new matron, therefore, had to deal with an undisciplined company of hard-bitten veterans who knew every trick of their poor trade, were left very much to their own devices, and had no inducement to take any interest in their work, and had little character either to gain or to lose. Sir Frederick's first introduction to Miss Luckes was momentary; he saw only a young and very pretty woman with a pleasant, kindly smile and most engaging manners. When asked what he thought of her, he replied that he could not conceive of anyone who appeared to be less suited for the post. How Miss Luckes altered that first impression and how by a steady scheme of reformation she succeeded in her great task is sympathetically described. Sir Frederick Treves' picture of nursing conditions in 1880 is a very black one and cannot, we think, be applied generally, although it is accurate enough as referring to a period of perhaps twenty years before.

